# Turkish validity and reliability of children’s environmental health knowledge and skills questionnaires for nursing students

**DOI:** 10.1186/s12912-025-02846-y

**Published:** 2025-02-27

**Authors:** Hatice Gürgen Şimşek, Şafak Dağhan

**Affiliations:** 1 Manisa Celal Bayar University, Manisa, Türkiye; 2 Ege University, Izmir, Türkiye

**Keywords:** Aptitude, Children, Environmental health, Knowledge, Psychometrics

## Abstract

**Background:**

A constantly changing and polluted environment can negatively affect children’s health. Nursing education should enable the training of future nurses who are fully equipped to protect and improve children’s health. In this respect, it is necessary to evaluate the knowledge and skills of nurse candidates. To examine the psychometric properties of the “Children’s Environmental Health Knowledge (ChEHK-Q) and Skills (ChEHS-Q) Questionnaire” among Turkish-speaking nursing students.

**Methods:**

This study employed a methodological method. An online survey was administered to 300 students in a nursing department in western Türkiye in 2021. Expert opinions were obtained for content and language validity. The validity of the scales was analysed via “Rasch measurement theory”, and whether the necessary preconditions for the analysis (the item function difference, local independence, reliability, and unidimensionality) were met was checked. Then, the item difficulty, person ability, and response threshold value ranking data were obtained. SPSS 25.0 software, LISREL Vrs. 8.80 program and Winsteps 3.92.1 Version program were used.

**Results:**

The content validity indices of both scales were above 0.80. Only item 12 of the ChEHS-Q was removed because it did not provide cultural appropriateness. Both scales met the Rasch measurement theory assumption and had model fit. The ChEHK-Q infit values ranged from 0.13 to 1.07, and the outfit values ranged from 0.88 to 1.17. The ChEHS-Q infit values ranged from 0.82 to 1.19, and the outfit values ranged from 0.86 to 1.19.

**Conclusions:**

Both questionnaires were found to be valid and reliable instruments in the Turkish language. Scales can provide ideas for shaping the nursing education curriculum in Türkiye. In this way, by determining which subjects are deficient, education programs can be arranged to eliminate these deficiencies. Nurses’ awareness of environmental health is also important in improving public health. Graduating nurses can inform families by identifying environmental risks and contribute to the creation of healthy living conditions.In addition, it can be used in clinical practice (public health nursing, etc.) and national and international research (randomized controlled trials evaluating planned educational activities).

**Clinical Trial Number:**

Not applicable.

**Supplementary Information:**

The online version contains supplementary material available at 10.1186/s12912-025-02846-y.

## Background

While our world is changing daily, many environmental changes seen on a global scale pose a challenge as well as a threat to human health [1]. Almost every child is exposed to environmental hazards at some point in their life [2]. These environmental exposures play a major role in children’s development [3, 4]. Children, including those in the foetal period, are at greater risk of environmental hazards than adults until adolescence. However, their exposure differs from that of adults [3]. Children are not small adults. Children, who have a special place in the global community and who will build our future, need healthy environments in which to grow, learn, and develop [3, 5]. Approximately 26% of the global population is younger than 15 years old, and this rate is higher in countries with low/middle income levels. In Türkiye, the population under the age of 15 is approximately 20% [5]. In 2016, approximately 1.6 million deaths among children under five years of age were environmentally friendly, almost two-thirds of which occurred in these countries [6]. The Sustainable Development Goals aim to eliminate preventable child mortality by 2030 [7]. If these targets are achieved, it is estimated that approximately 10 million lives under the age of 5 will be saved [8]. The environment, which is one of the basic concepts of the nursing profession, has an important role in child health [6, 9]. Nurses are conscious of the impacts of environmental degradation on health [10]. However, International Council of Nurses call for nurses to take action on local and global environmental health issues that affect health [11]. Recently, the issue of child environmental health has attracted increasing attention in nursing research [12, 13]. Reports published at the global level also justify the interest in this issue [14, 15]. The effects of many substances on health, whose use in daily life is constantly increasing or newly produced, are also a matter of curiosity [16]. “Child Environmental Health” is a scholarly field that “examines how environmental exposures (chemical, nutritional and social) early in life affect health and development during childhood and even throughout human life”. Its focus is the prevention, diagnosis and treatment of diseases in children related to environmentally detrimental exposures [17].

Notably, health workers can be strong advocates in addressing children’s environmental health [11, 18, 19]. To take action, they need to generate evidence and provide clear recommendations for remedial/preventive activities [20]. However, environmental health does not have the place it deserves in the routine practices of healthcare experts [21, 22]. These studies emphasize that environmental risk factors are not questioned, and the need for training healthcare workers on environmental history is emphasized [21–23]. In addition, studies have shown that nurses feel uncomfortable at the point of discussing possible exposure sources related to the disease with the patient and family [22]. The reason for this discomfort can be considered insufficient knowledge and skills on the subject. However, American Nurses Associations (ANA) has set competences in the field of environmental health for undergraduate nurses. It states that they communicate with healthcare recipients, families, and the community to identify environmental health risks [20]. In the literature, the knowledge, skills, attitudes, and competency necessary to be possessed by undergraduate nurses in the fields of the environment and child health are summarized [18]. In our country, there are descriptions connected to the notion of the environment in ‘The Nursing Undergraduate Education Programme National Qualifications’ and the target learning outcomes [9]. All these can be seen as very good bases for equipping future nurses in this field. However, in the studies carried out, unfortunately, nursing education cannot educate students sufficiently on this subject, and deficiencies in terms of knowledge and skills connected to child environmental health are expressed [12, 21]. It is clear that future nurses need to be supported in this regard, and measurement tools are needed to determine whether educational goals are achieved. In the literature, tools for measuring nursing students’ knowledge and skills related to child health and the environment have been developed and these tools are valid and reliable in various countries [13, 24–26]. ANA also recommends the use of assessment tools to identify environmental hazards [20]. However, when studies conducted with nursing students in Türkiye were examined, it was determined that there were no accessible measurement tools and study findings that simultaneously assessed multiple levels of knowledge about environmental risk factors (pesticides, mercury, lead, etc.) specifically for children. Besides that for undergraduate students in Türkiye, “creating a safe environment; participating in social responsibility projects related to the effects of the environment on human health” is listed as a basic skill at skill level 3 [9]. Qualitative study findings examine students’ self-perceptions of talent/skill during home visits conducted within the scope of public health nursing field practice in Türkiye [27, 28]. However, the lack of measurement tools that can be used in the measurement of these skills and knowledges especially in the field of paediatric environmental health, in the literature of Türkiye reveals the need to complete this field. The existence of structured measurement tools in the international literature that can simultaneously assess the knowledge and skills of nursing students regarding many environmental exposures related to child health and various environments (home, school, playground) can be considered an advantage [25, 26]. The study aimed to adapt the “Children’s Environmental Health Knowledge (ChEHK-Q) and Skills (ChEHS-Q) Questionnaires” into Turkish and to appraise its psychometric characteristics [25].

Research questions;

1. Is the Turkish version of ChEHK-Q valid and reliable?
2. Is the Turkish version of ChEHS-Q valid and reliable?

## Methods

### Type of study

This type of study is a methodological study.

### Place/sample of the study

The study’s sample group consisted of nursing students at a university in western Türkiye. It is recommended that 300 or more samples be taken to reveal the factor structure of a test while the scales are validated [29]. Based on this this recommendation, 300 nursing students were involved. The pilot study was conducted with 10 students not included in the sample, and the study was conducted with 310 students (*n* = 310). Nursing students who were over 18 years of age, who were continuing their education actively, and who volunteered were included. Nursing students who were under 18 years of age, did not volunteer to participate in the study, and had suspended their registration were excluded from the study. Online surveys via Google Forms were used to gather study data between March and July 2021. The first researcher included the online class groups and invited the students one by one to participate in the research, and motivated them to participate. Data collection started with the 4th grade, and as positive/negative responses were received from the entire class, the next grade was moved on. The time to answer the survey was approximately 10–15 min.

### Data collection forms

The following forms were used to collect the research data:

- “*Demographic characteristics question form*”: There are four questions: age, gender, class, environment and participation in a course/conference/seminar related to nursing.
- *“Children’s Environmental Health Knowledge Questionnaire (ChEHK-Q)*”: It consists of 26 items and has three answers: “True/False/Do not know”. To calculate the general knowledge score, one point is added to the correct answers for each item. Items 1, 3, 6, 8, 9, 11, 12, 14, 15, 16, 18, and 22 are correct, whereas the others are incorrect. The maximum score is 26 (knowledge index 100%). Several indices can be derived: the “knowledge index (overall score/26 × 100) and the ignorance index (number of ‘do not know’/26 × 100)”. The knowledge levels are categorized as follows: ‘Excellent knowledge level (> 90% correct answers), very good knowledge level (90–80% correct answers), good knowledge level (80–60% correct answers), inadequate knowledge level (60–40% correct answers), and poor knowledge level (< 40% correct answers)’. The Cronbach’s alpha of the original Spanish form is 0.98 [26].The original name was renamed the “Child Environmental Health Relationship Knowledge Scale” in terms of cultural meaning during the adaptation process.
- “*Children’s Environmental Health Skills Questionnaire (ChEHS-Q)*”: There are 12 items on a 5-point Likert scale. Each item is evaluated with values ranging from “1 (strongly disagree) to 5 (strongly agree)”. The overall score is computed by “adding the points that each student assigns to each item”. A minimum score of 12 points and the highest score of 60 points can be obtained. The skill index is calculated as follows: skill index = total points/60 × 100. Skill levels are categorized as follows: ‘Excellent skill level (> 90% perceived skills), very good skill level (90–80% perceived skills), good skill level (80–70% perceived skills), poor skill level (70–50% perceived skills), and poor skill level (< 50% perceived skills)’. The Cronbach’s alpha of the original Spanish form is 0.87 [26]. The original name was renamed the “Child and Environmental Health Relationship Skills Scale” in terms of cultural meaning during the adaptation process.

### Cultural adjustment of questionnaires

The adaptation of the scales (English versions) into Turkish was carried out in four stages [25, 30]:

1. The scales were translated into Turkish by four nurse academicians who were proficient in English and Turkish. These translations were evaluated by the researchers in terms of language meaning and concept appropriateness, and a Turkish scale draft was created. The scale drafts were back-translated by two translators who were native English speakers, were proficient in Turkish and English, were independent of each other, had no knowledge of the scales, and were subsequently compared with English scales [30].
2. The Turkish and English versions were evaluated by language and content validity by five nurse faculty members (experienced in paediatrics, public health, and nursing education), who are experts in their fields with good command of English. The experts rated the comprehensibility of the Turkish scale items with the Davis technique on a scale of 1–4 points: “1 point = not appropriate, 2 points = somewhat appropriate, 3 points = appropriate, 4 points = completely appropriate” [31]. Content validity indices were calculated as follows: “the item-based content validity index (I-CVI) was calculated by dividing the number of experts who gave 3 or 4 points to each item by the total number of experts,” and “a scale-based content validity index (S-CVI) was determined with the average ratios of the items” [32]. The comprehensibility of each item is regulated according to the recommendations of the experts. Since the ‘Pediatric Environmental Health Specialty Units’ mentioned in the 12th question of the ChEHS-Q are not available in Türkiye and there is no other unit suitable for this unit, this item was removed from the scale (“Item 12: I do NOT feel able to do my job as a nurse in a Pediatric Environmental Health Specialty Unit.”).
3. For the pilot application, the scale forms were given to 10 nursing students who showed sample characteristics. Two students reported that “Item 7” was not fully understood in ChEHK-Q and that the expression for increased humidity in the house had been edited. Data from pilot applications were excluded from the analysis.
4. Finally, the scales were applied to a research sample of 300 people.

### Psychometric test stages

The reliability and validity of the ChEHK-Q and ChEHS-Q were analysed according to Rasch measurement theory [33]. In Rasch measurement theory, respondents and items in a scale can be positioned on the same scale. The equation of item response theory, which is valid for a one-parameter logistic model, is used to calculate “the probability that a respondent at ability level **θ** will correctly answer item **i** at difficulty **b**_**i**_” [34–36].

In Rasch analysis, first, the response method of the items in the test is examined, and the best fit mathematical Rasch model is selected. The “Andrich Rating Scale Model or Masters’ Partial Credit Model” is widely used when the response category of the items has three or more options. In this study, the Dichotomous Rasch Model for the ChEHK-Q and the Partial Credit Model for the ChEHS-Q were used to investigate model fit, internal construct validity, and reliability [36]. In Rasch analysis, these criteria must be fulfilled [36, 37]:

- *Unidimensionality*:This is the first assumption. A model consisting of one dimension was defined for both scales, and confirmatory factor analysis (CFA) was conducted to determine whether these hypothetical models fit the model data. In addition, the principal component analysis results of the residuals from the Rasch analysis outputs were examined to determine whether the scale had a one-dimensional structure. This method predicts that there should not be different relationships between items that indicate another dimension other than random relationships. Therefore, the ‘Contrasts’ obtained as a result of the analysis, which correspond to the residual variances outside the variance that can be explained by the model, were analysed. The ratio of the eigenvalue of the unexplained raw variance in Contrast 1 (1.56), which has the highest eigenvalue for ChEHK-Q, to the eigenvalue of the unexplained raw variance with the Rasch model (26.0) was found to be 0.07. Since this ratio is less than 1/3 (~ 0.33), the ChEHK-Q is unidimensional (it measures a single latent construct). A total of 32.7% of the total variance explained by the Rasch model comes from the measurements. The remaining 67.3% variance indicates the coincidental relationships in the measurements and indicates that there is no other significant dimension other than the characteristic measured by the scale. For the ChEHS-Q, the 1st contrast value of the unexplained variance was found to be 1.98, and since this eigenvalue was < 2, the items in the test corresponded to a single dimension, and the variance unexplained by the model did not point to another dimension. The ratio of the eigenvalue (1.98) of the unexplained raw variance in the 1st contrast with the highest eigenvalue to the eigenvalue (11.0) of the raw variance unexplained by the Rasch model was found to be 0.18, and since this ratio is less than 1/3 (~ 0.33), the ChEHS-Q is unidimensional; in other words, it measures a single latent construct. A total of 32.6% of the total variance explained by the Rasch model comes from the measurements. The remaining 67.4% of the variance represents random relationships in the measurements and indicates that there is no other significant dimension other than the characteristic measured by the scale [36, 37].
- *Local independence*: This is one of the most fundamental assumptions. The response of each scale item should be independent of all other items along the ability continuum. When the assumption is not met, the unidimensionality of the scale is affected. For this reason, it was examined whether the inter-item residual correlations of the items of the ChEHK-Q and ChEHS-Q were below *r* ≤ 0.32. On the other hand, negative correlation coefficients do not make sense since the relationships between the related items are inversely related; negative correlation coefficients were excluded from the evaluation. For ChEHK-Q, no value exceeded 0.12. For the ChEHS-Q, the highest positive correlation between the standardized residuals was 0.30. The assumption of local independence was met for both scales [36, 37].
- *Differential Item Functioning (DIF):* The fact that the measures contain DIF may also affect the data-model fit. When respondents in different groups (e.g., gender) within the sample who have equal ability levels in terms of the construct measured respond differently to an item, this indicates that the item contains DIF [36]. If DIF is observed, systematic error is introduced into the measurements, and the validity of the scale is lower than it should be. In this study, it was investigated whether the items of both scales contained DIF according to gender. The contrast of the between-group DIF measures of the ChEHK-Q was between − 0.65 and 0.98 logit values and was not statistically significant, and the probability values corresponding to the Mantel–Hanzel chi-square values of *p* ≥ 0.03 were interpreted as indicators that the items did not contain DIF [35, 38]. Similarly, the fact that the contrast of the ChEHS-Q’s between-group DIF measures was between − 0.19 and 0.28 logit values and was not statistically significant and that the probability values corresponding to the Mantel–Hanzel chi-square values were *p* ≥ 0.03 were interpreted as indicators that these scale items did not contain DIF [35, 38]. The difficulty levels of the items of both the ChEHK-Q and the ChEHS-Q do not differ significantly according to the gender of the individuals. In other words, the items in the tests did not contain item functions that varied according to participants from the same population but belonged to different subpopulations (e.g., male‒female). The results obtained in future applications at both scales should be interpreted by taking this situation into consideration. In this respect, DIF analyses provide evidence on whether the scales have cross-validation.
- *Model fit*: Fit statistics of the data-model fit were evaluated for both scales. The log-likelihood chi-square value = 7821.89 for ChEHK-Q, with approximately 7831 degrees of freedom and *p* = 0.53. The log-likelihood chi-square value = 8163.31 for ChEHS-Q, with approximately 8714 degrees of freedom and *p* = 0.53. These statistics support the data-Rasch model fit. In addition, the item-fit residual values were in the range of ± 2.5, indicating that both scales fit the Rasch model well [35].
- *Reliability*: For reliability analysis, values for items and subjects were calculated, and values of 0.80 and above were considered good. The model also considers the separation index, “which indicates whether respondents answered every question in the same rating group and refrained from responding in categories at either end of the scale.” Ideally, separation values should be greater than 2. In this study, the reliability coefficient of the ChEHK-Q in the Rasch model analysis was 0.76 (separation index = 1.86) for the subjects and 0.92 (separation index = 5.80) for the items. The reliability coefficients for the ChEHS-Q were 0.79 (separation index = 1.97) for the subjects and 0.94 (separation index = 4.09) for the scale. The items are enough to categorize students according to their knowledge and skills. For both scales, the number of people in the sample is very close to 2, and the person reliability is approximately 0.80, which can be considered a limitation [35].

For the scales that met all these assumptions, item difficulty and person ability, response threshold value ranking data were then evaluated [36, 37].

### Statistical analyses

For the data analysis, SPSS 25.0 software was used for descriptive data, the LISREL Vrs. 8.80 program for CFA [39], the Winsteps 3.92.1 Version program was used for Rasch analyses. The number, percentage, minimum and maximum values were calculated for descriptive statistics. Goodness of fit indexes were presented as the goodness of fit index (GFI), adjusted goodness of fit index (AGFI), comparative fit index (CFI), root mean square error of approximation (RMSEA), and standardized root mean residual (SRMR) [40].

### Ethical considerations

The appropriate author granted permission for the scale to be used. Ethics committee permission (Date: 03.02.2021, Number: 20.478.486/743) and institutional permission from the faculty (Date: 17.03.2021, Number: E.43954) were received. Before the questions appear at the beginning of the online survey, each participant provided informed consent.

## Results

### Demographic characteristics

The participants’ average age was 21.85 ± 1.41 years (min: 19, max: 33). A total of 78.0% (*n* = 234) of the participants were female, 22.0% (*n* = 66) were male, 47.3% (*n* = 142) were in 4th grade, 46.0% (*n* = 138) were in 3rd grade, and 6.7% (*n* = 20) were in 2nd grade. A total of 31.0% (*n* = 93) of the students stated that they had attended a course/conference/seminar related to the environment and nursing, whereas 69.0% (*n* = 207) stated that they had not.

## Children’s environmental health knowledge questionnaire (ChEHK-Q)

### Content validity

The S-CVI was 0.96, whereas the I-CVI was between 0.80 and 1.00. The scale did not contain any items that were removed.

### Construct validity

A model consisting of 26 items and one dimension was defined with the data obtained from the pretest application of the ChEHK-Q, and this hypothetical model was examined via CFA. Accordingly, the fit chi-square value is 287.13, the degree of freedom is 249, and *p* = 0.53. The other goodness-of-fit indices were GFI = 0.93, AGFI = 0.92, CFI = 1.00, RMSEA = 0.019, and SRMR = 0.046.

### Rasch analyses

Table 1 shows the fit values for individual items and all items. The outfit values ranged from 0.88 to 1.17, and the infit values ranged from 0.13 to 1.07. The item difficulty values ranged from − 2.97 (Item 1, the easiest item) to 3.19 (Item 19, the most difficult item).

**Table 1:** Item fit statistics of the items of the Children’s Environmental Health Knowledge Questionnaire (*n* = 300)

Items	Difficulty	SE^a^	Infit^b^	Outfit^c^
1. “The pediatric population is more susceptible to environmental threats due to their biological immaturity.”	-2.97	0.23	0.98	0.94
2. “The increased energy and metabolic consumption of the pediatric population protects children from environmental hazards.”	0.29	0.13	1.03	0.99
3. “The higher rate of cell growth during the pediatric age increases the risk of health effects caused by environmental factors.”	-0.09	0.13	0.98	1.06
4. “Environmental factors do not influence hormonal secretion during puberty.”	-2.53	0.20	1.12	1.01
5. “Nitrogen oxide from fossil fuels in the home and tobacco smoke causes redness and burns on the skin.”	2.59	0.21	0.99	0.83
6. “Particles from animals exacerbate asthma crisis.”	-1.36	0.14	1.01	1.17
7. “Increased humidity at home improves respiratory diseases in children.”	-0.38	0.13	0.98	0.99
8. “Passive smoking is associated with the development of acute leukemias in children.”	0.52	0.13	0.99	0.97
9. “Childhood leukemia incidence rates are higher in the areas most exposed to radon.”	0.59	0.13	1.02	1.00
10. “Overexposure to solar ultraviolet radiations can damage the skin of adults more severely than that of children.”	0.50	0.13	0.94	0.88
11. “During childhood more than half of the expected lifetime solar ultraviolet radiation is absorbed.”	0.99	0.14	1.01	0.98
12. “Lead accumulates in the body affecting the nervous system.”	-1.87	0.16	1.07	1.07
13. “Chronic dietary exposure to mercury (fish and shellfish) is less toxic to children’s central nervous system than to adults.”	0.32	0.13	0.96	0.91
14. “Exposure to pesticides increases the risk of developing attention deficit problems in school-aged children.”	0.32	0.13	1.02	1.09
15. “Children born to smoking mothers during pregnancy are at risk of lower intellectual capacity.”	-0.95	0.14	0.93	0.89
16. “Exposure to organic solvents during fetal development can cause learning disabilities in children.”	-0.72	0.13	0.96	1.02
17. “Water containing nitrates can only cause intoxication during childhood.”	0.21	0.13	0.95	0.89
18. “Chlorination of water forms subproducts from the disinfection process that have been classified as carcinogenic.”	0.57	0.13	0.96	1.01
19. “The major source of childhood exposure to pesticides is through ambient air.”	3.19	0.26	1.00	1.06
20. “The main route of exposure to mercury is through cereal intake.”	-0.04	0.13	1.00	0.98
21. “Exposure to lead through diet occurs mainly through fish intake.”	1.67	0.16	1.00	0.90
22. “Food colorings and preservatives are associated with central nervous system problems.”	0.01	0.13	1.05	1.11
23. “Genetically modified foods cause fewer allergic reactions in children.”	-1.48	0.15	1.04	1.09
24. “Schools and nurseries are environmentally safe places.”	1.34	0.15	1.01	1.11
25. “Children are exposed to higher concentrations of air pollutants at home than outdoors.”	-0.51	0.13	1.03	1.00
26. “Parks and gardens are the areas with the least environmental pollutants where children can play.”	-0.23	0.13	0.99	0.99

^a^SE = standard error of item difficulty values, ^b^Infit = weighted mean square fit, ^c^Outfit = unweighted mean square fit

Note: The maximum acceptability is between 0.5 and 1.5

**Fig. 1:**
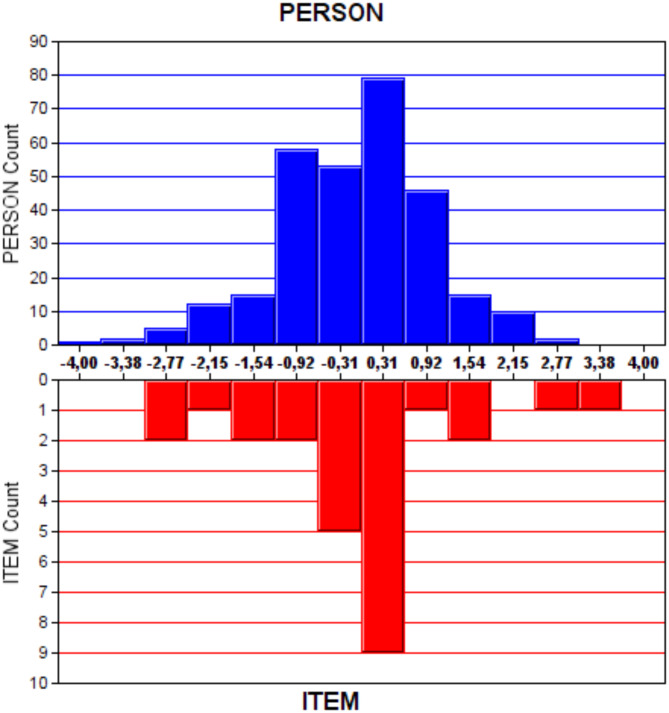
Distribution of Item Difficulty Levels and Frequencies of Participant Scores for the ChEHK-Q

The difficulty levels of the items in the scale corresponding to the ability continuum and the frequencies of the ability scores of the participants in the same ability continuum are shown in the histogram given in Fig. 1. At least one item that can be measured at each ability level is present in the ChEHK-Q. On the other hand, some of the scale items are clustered at the − 1.0 and 1.0 difficulty levels.

### Children’s environmental health skills questionnaire (ChEHS-Q)

### Content validity

The S-CVI was 0.95, whereas the I-CVI was between 0.80 and 1.00. Item 12 was removed because of a lack of cultural appropriateness.

### Construct validity

A model consisting of 11 items and one dimension was defined with the data obtained from the pretest application of the ChEHS-Q, and this hypothetical model was tested for model data fit by performing CFA. Accordingly, a Model Fit Chi-Square value = 103.83, degree of freedom = 41 and *p* = 0.00 were obtained. The goodness-of-fit indices were GFI = 0.94, AGFI = 0.90, CFI = 0.94, RMSEA = 0.072 and SRMR = 0.060. According to the CFA results, the standardized factor loadings (item validity coefficients) of the items are between 0.43 and 0.58. The R^2^ (item reliability) values of the items are between 0.14 and 0.38. T values show that the estimated factor loadings are significant at the *p* ≤ 0.01 level for 11 items.

### Rasch analyses

Table 2 shows the fit values for each item. The infit values ranged from 0.82 to 1.19, and the outfit values ranged from 0.86 to 1.19. The item difficulty values varied between − 0.56 (Item 6, the easiest item) and 0.54 (Item 3, the most difficult item).

**Table 2:** Item fit statistics of the items of the Children’s Environmental Health Skills Questionnaire (*n* = 300)

Items	Difficulty	SE^a^	Infit^b^	Outfit^c^
1. “I am able to assess the main environmental risks to which a child is exposed.”	0.02	0.07	0.82	1.01
2. “I am NOT able to identify the environmental risks that can cause respiratory diseases in a child.”	-0.03	0.07	1.11	1.14
3. “I am able to identify the environmental risks that can cause neoplastic diseases in a child.”	0.54	0.06	0.87	0.86
4. “I am NOT able to identify the environmental risks that can cause neurological disorders in a child.”	0.29	0.06	1.02	1.06
5. “I am able to provide health education to parents about the main contaminants in their child’s food.”	0.25	0.06	0.95	0.95
6. “I am NOT able to identify the environmental risks in playgrounds.”	-0.56	0.07	1.17	1.16
7. “I am able to provide health education to parents about actions to minimize environmental risks to which a child is exposed when playing outdoors.”	-0.14	0.07	0.95	1.02
8. “I am NOT able to identify the environmental risks in a child’s home.”	-0.22	0.07	1.06	1.07
9. “I am able to provide health promotion to parents about environmental risks at home.”	0.08	0.07	0.86	0.87
10. “I am able to identify the environmental risks in a child’s school.”	-0.12	0.07	1.19	1.19
11. “I am NOT able to identify the actions needed to combat environmental risks in a child’s school.”	-0.10	0.07	1.07	1.08

^a^SE = standard error of item difficulty values, ^b^Infit = weighted mean square fit, ^c^Outfit = unweighted mean square fit

Note: The maximum acceptability is between 0.5 and 1.5

**Fig. 2:**
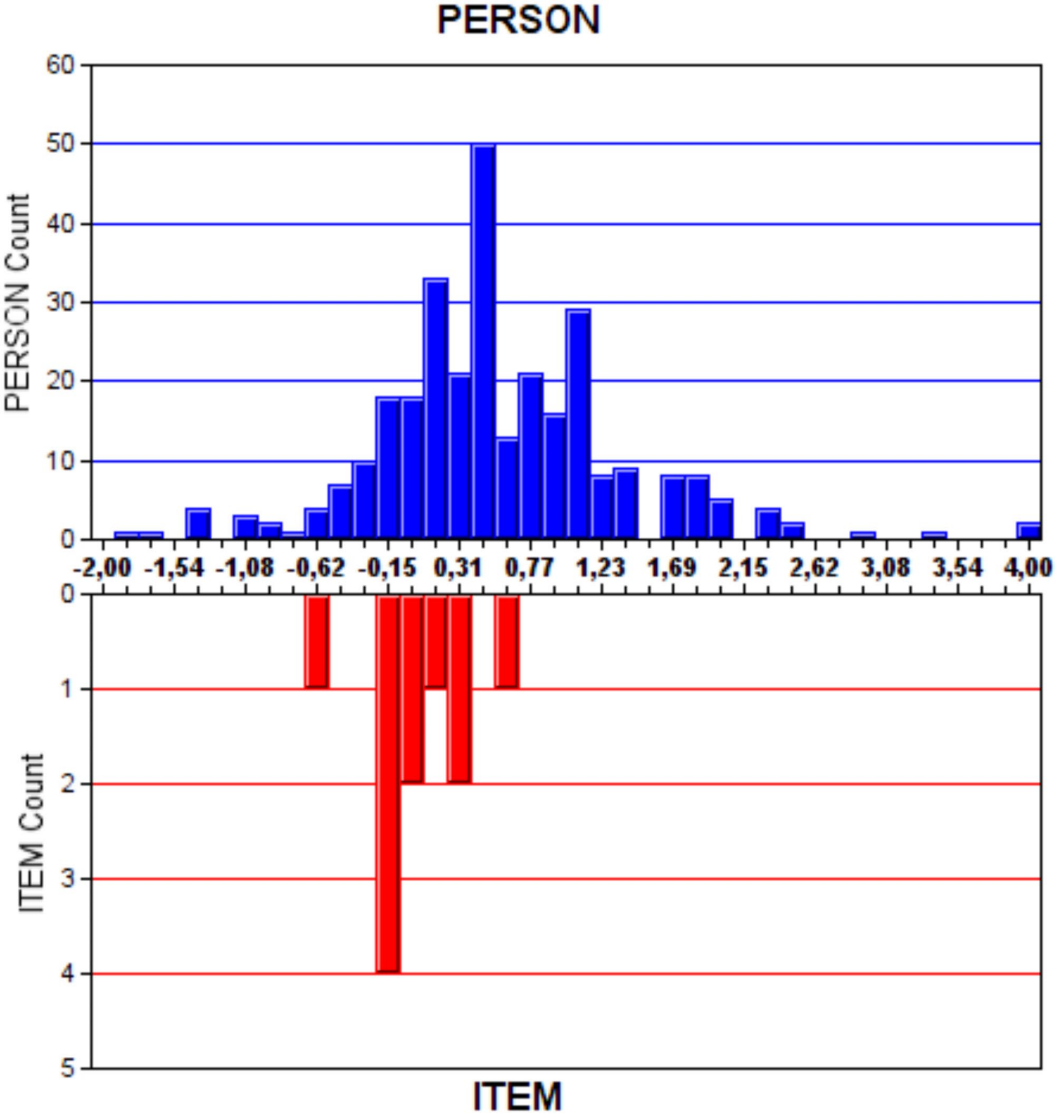
Distribution of Item Difficulty Levels and Frequencies of Participant Scores for the ChEHS-Q

The corresponding difficulty levels of the items in the scale on the ability continuum and the frequencies of the participants’ ability scores on the same ability continuum are shown in Fig. 2. Most scale items were concentrated at the difficulty level of -0.5-0.5.

The ChEHS-Q Likert-type scale has thresholds that range from a low of -1.28 to a high of 1.68, as Table 3 illustrates.

**Table 3:** Threshold values showing transitions to response categories based on Scale

Likert category	Andrich Threshold Values-Logit (SE)^a^	INFIT	OUTFIT
0	-		
1	-1.28 (0.09)	1.04	1.06
2	-0.67 (0.05)	0.86	0.91
3	0.27 (0.04)	0.94	0.93
4	1.68 (0.05)	0.94	0.95

^a^Standard error

## Discussion

Using standard measurement tools at the global level provides many benefits in determining the situation and progress by ensuring a common language among healthcare professionals. It is important to measure the knowledge and skills of Turkish nursing students with respect to child environmental health, and studies conducted in various countries using samples of nursing students may facilitate comparisons [24–26].

The validity and reliability of scales can be tested with different models. In the Rasch model, an attempt is made to estimate the probability of what an individual at a certain ability level can do against a task that is requested to be performed [41]. It assumes that “the probability of a person passing an item is related to the person’s ability and the difficulty of the item” [34, 36]. Research findings suggest that this analysis, which is commonly used in the life sciences, is less common in nursing and that its reporting should be more systematic. To increase the quality and transparency of reporting, it is important to follow the minimum standards recommended in reporting the analysis in this study in terms of contributing to the literature [42]. In this study, the data met the necessary conditions for Rash analysis [36, 37]. Only the fact that the person separation indices for both scales were very close to 2 can be considered a limitation. This finding was found to be one-to-one similar to the original scales, while the Portuguese version showed similarity only for the ChEHK-Q [24, 26]. This situation can be interpreted in the literature, as the tool may not be sensitive enough to separate high performers from low performers. More items may be needed at this point [35]. In China, these values were determined to be much higher than recommended [13].

The findings of this study support that both the ChEHK-Q and the ChEHS-Q have a unidimensional structure, as in the original and other cultures, and that factorial construct validity was achieved [13, 25, 26]. In the Portuguese version, it was suggested that the findings did not allow for explaining the construct validity for both scales, but they should be considered as one-dimensional [24]. When the model-data fit indices and error indices in CFA were considered together, the model-data fit of the tested data was good for both scales [43–45].

Model fit parameters can be calculated via Rasch analysis. For a good item fit (infit-outfit), there must be closeness between the observed and expected data. In regard to items that are near the potential trait level of participants, anomalous response patterns can be found via infit, an adjusted statistic with weighted data. The participants’ latent trait is considerably less susceptible to things than the outfit trait, which is an adjustment statistic. With a center value of 1, the infit and outfit span from 0 to positive infinity, indicating that the model fully fits the real data [35]. In this study, the model infit and outfit values of both the ChEHK-Q and the ChEHS-Q were not greater than the maximum acceptability limits of 0.5–1.5, indicating that all the items fit the model well [35]. In the literature, there are differences in the limit values (Spain: 0.8–1.2; England/China/Portugal: 0.7–1.3) considered [13, 24–26]. The infit and outfit values in this study did not exceed all these specified limits, similar to those in England, China, and Portugal [13, 24, 25].

Rasch analysis also determines each item’s difficulty. The average degree of item difficulty is 0, and difficulty is defined as “the value of the latent feature required for a participant to have a 50% likelihood of endorsing the item” [35]. In this study, the easiest item of the ChEHK-Q was determined to be “Item 1”, whereas the most difficult item was determined to be “Item 19”, which is the same as studies conducted in England and Spain [25, 26]. In studies conducted with community nurses in China and nursing students in Portugal, the easiest item was again “Item 1”, and the most difficult item was “Item 5” [13, 24]. It can be interpreted that these differences between countries may be due to differences in sample groups (nursing students, public health nurses) and curriculum differences. The easiest item to answer is that nursing education provides nursing students and therefore nurses with a perspective on the level of awareness related to children’s development and their sensitivity to environmental risks. However, when looking at difficult items, it is clear that they require a knowledge load related to environmental health to be answered. ANA also emphasized the necessity of environmental health concept knowledge for nursing practice, and it was determined that undergraduate nurses should be able to communicate about environmental health risks as a competency [20]. In Türkiye, under the leadership of public health nursing, joint curriculum recommendations can be made by identifying the missing points in the curriculum and holding sessions with the Delphi technique to complete them. The easiest item of the ChEHS-Q is “Item 6”, and the most difficult is “Item 3”. In studies conducted in China and England, the easiest item was “Item 8”, whereas the most difficult item was “Item 3”, which is consistent with the study findings [13, 25]. In Portugal, the easiest item was “Item 2” and the most difficult item was “Item 3” [24]. The change in the easy items in all studies can be attributed to the nursing education curricula (theoretical and practical) of the countries. However, it should be considered as a remarkable finding that the most difficult item in all studies was “Item 3” [13, 24, 25]. These results can be interpreted as nurses and nursing students being more familiar with and working on environmental risks in areas such as school or home environments. However, deficiencies in knowledge should be mentioned first when determining the environmental risks of neoplastic diseases. Since the literature also supports this interpretation, epidemiological studies provide important evidence on this subject, but this information is not included in the daily practices of clinicians who tend to affect children affected by this illness [22, 46]. A previous study revealed that nurses were more uncomfortable discussing sources of exposure to neoplastic diseases with patients and their families than physicians were [22]. To eliminate this discomfort, it is important for future nurses to enter the prepared workforce. Considering the most difficult and easiest items of both scales can provide an idea of which points need to be developed in the nursing education program. It may be recommended that revisions be made to the curriculum, taking into account the recommendations of leading organizations on child environmental health.

To assess the functioning of the items in Rash analysis, item maps are considered. The participants’ latent characteristics (θ) are compared with the item difficulty level (β). The closer the distribution of item difficulties is to the persons’ latent trait distributions, the higher the test’s accuracy is considered [35]. In this study, the majority of the students and the majority of the items on the item map were located in the central area. This implies that the variety of test items given to students was suitable for this response group. There was at least one item that could be measured at each ability level for both scales. The findings of this study align with the literature [13, 24, 25].

Rasch analysis calculates the item threshold or the distance between each item’s neighboring choices. The thresholds for ChEHS-Q must shift from negative to positive, and a shift in this sequence could mean that the response categories are not operating as they should [47]. The threshold values increased simultaneously as the ability level increased in the response categories of the ChEHS-Q items from the lowest to the highest. In accordance with the literature, the five response options of the Likert-type scale worked correctly [13, 24, 25].

In the nursing education curriculum in Türkiye, especially the public health nursing and child health nursing courses should be strengthened by taking into account the child health-environment interaction. Topics on the effects of the environment on health can be integrated into the curriculum to increase students’ knowledge of child environmental health. More opportunities can be provided for the transfer of theoretical knowledge to the clinic. For this purpose, case discussions can be held that include different application environments (school, home, playground), and students can be included in field studies to gain practical knowledge. the ChEHK-Q and the ChEHS-Q, measure student nurses’ knowledge and skills in identifying, assessing, and managing environmental health risks to children. Case studies, simulations, and practical training can be organized in training programs to help students gain this knowledge and skills. While doing all of this, it may be recommended to use educational technologies (online education, video, etc.) and encourage students to do research on this subject.

## Conclusion

According to the research results, the Turkish versions of both the ChEHK-Q and the ChEHS-Q met the necessary assumptions for the Rasch Model (fit, unidimensionality, local independence required by the Rasch model), and none of the items contained DIF. Reliability (item/person) levels were high on an item basis and close to the limit on a person basis; the scales were reliable and had internal construct validity. The structure of the ChECK-Q, which consists of 26 items, and the ChEHS-Q, which consists of 11 items, were validated. Turkish versions of the ChEHK-Q and ChEHS-Q can be used to define the current knowledge and skills of nursing students and primary health care workers (nurses, midwives) on this subject. Scales can be tested on different samples (pediatric nurses and midwives) serving in the field of child health. If Pediatric Environmental Health Specialty Units are established in Türkiye, it may be recommended to improve the scale by adding “Item 12” to the Turkish version of ChEHS-Q. In addition to this, it may be recommended that the exposures of vulnerable groups, especially children, be clearly defined under the heading of “environment” in the updating stages of national nursing curricula. Also, the effects of lessons/planned and structured training about children and the environment on the formation of knowledge and skills can be evaluated.

This study has several limitations. Because participation was voluntary, the study results may be prone to self-selection bias. This may imply that participants may have been those most interested in environmental issues, for example, compromising the generalizability of the findings. Additionally, the use of self-administered surveys may increase the risk of desirability bias (e.g., respondents responding to provide a positive impression of themselves) or other response biases (e.g., demand attributes). Online data collection may cause participants to reflect personal or socially accepted truthful views. They may give answers that are more acceptable in an online environment. However, since the data were collected online, test‒retest evaluation could not be performed.

## Electronic supplementary material

Below is the link to the electronic supplementary material.

Supplementary Material 1

## Data Availability

Data is provided within the manuscript or supplementary information files.

## Abbreviations

ANA: American Nurses Associations
CFA: Confirmatory factor analysis
ChEHK-Q: Children’s Environmental Health Knowledge Questionnaire
ChEHS-Q: Children’s Environmental Health Skills Questionnaire
I-CVI: Item-based content validity index
S-CVI: Scale-based content validity index
DIF: Differential Item Functioning
GFI: Goodness of fit index
AGFI: Adjusted goodness of fit index
CFI: Comparative fit index
RMSEA: Root mean square error of approximation
SRMR: Standardized root mean residual
