# Supplementary material for: Turkish validity and reliability of children’s environmental health knowledge and skills questionnaires for nursing students
Source: BMC Nurs. 2025 Feb 27;24:226. doi: 10.1186/s12912-025-02846-y (PMC11869414; doi:10.1186/s12912-025-02846-y)
Supplement: Supplementary file 1 — Supplementary Material 1 [file 12912_2025_2846_MOESM1_ESM.docx]

**ÇOCUK ÇEVRE SAĞLIĞI BİLGİ ÖLÇEĞİ ANKETI**

| **Items** | **Doğru** | **Yanlış** | **Bilmiyorum** |
| --- | --- | --- | --- |
| 1. Çocuklar, biyolojik gelişimlerini tamamlamadığı için çevresel tehlikelere karşı daha hassastır. |  |  |  |
| 2. Çocukların artan enerji ve metabolik tüketimi, onları çevresel tehlikelerden korur. |  |  |  |
| 3. Çocukluk çağında daha yüksek oranda olan hücre büyümesi, çevresel faktörlere bağlı gelişebilecek sağlığı etkileyen riskleri arttırır. |  |  |  |
| 4. Çevresel faktörler ergenlik döneminde hormon salgılanmasını etkilemez. |  |  |  |
| 5. Evdeki fosil yakıtlardan kaynaklanan nitrojen oksit ve tütün dumanı, ciltte kızarıklığa ve yanmaya neden olur. |  |  |  |
| 6. Hayvanlardan yayılan partiküller astım krizlerini ağırlaştırır. |  |  |  |
| 7. Evdeki artmış nem, çocuklarda solunum yolu hastalıklarını iyileştirir. |  |  |  |
| 8. Çocuklarda pasif sigara içiciliği akut lösemi gelişimi ile ilişkilidir. |  |  |  |
| 9. Radon gazına maruz kalan bölgelerde çocukluk çağı lösemisi görülme sıklığı daha yüksektir. |  |  |  |
| 10. Güneşin ultraviyole ışınlarına aşırı maruz kalma, yetişkinlerin cildine çocuklarınkinden daha ciddi bir şekilde zarar verebilir. |  |  |  |
| 11. Ömür boyu alınması beklenen güneş kaynaklı morötesi radyasyonun yarısından fazlası çocukluk döneminde emilir. |  |  |  |
| 12. Vücutta kurşun birikmesi, sinir sistemini etkiler. |  |  |  |
| 13. Sürekli olarak beslenme yoluyla civaya maruz kalma (balık ve kabuklu deniz hayvanları) çocukların merkezi sinir sistemine yetişkinlerden daha az toksik etki yapmaktadır. |  |  |  |
| 14. Pestisitlere maruz kalma, okul çağındaki çocuklarda dikkat eksikliği sorunlarının gelişme riskini artırır. |  |  |  |
| 15. Hamilelik döneminde sigara içen annelerin çocuklarının entelektüel kapasitelerinin daha düşük olma riski vardır. |  |  |  |
| 16. Fetal gelişim sırasında organik çözücülere maruz kalmak çocuklarda öğrenme güçlüğüne neden olabilir. |  |  |  |
| 17. Nitrat içeren su, sadece çocukluk döneminde zehirlenmeye neden olabilir. |  |  |  |
| 18. Dezenfeksiyon sürecinde suyun klorlanmasından ortaya çıkan alt ürünler kanserojen olarak sınıflandırılır. |  |  |  |
| 19. Çocukluk döneminde pestisitlere maruz kalmanın ana kaynağı ortam havasıdır. |  |  |  |
| 20. Civaya maruz kalmanın ana nedeni, tahıl/mısır gevreği tüketimidir. |  |  |  |
| 21. Beslenmeye bağlı kurşuna maruz kalma çoğunlukla balık tüketimi ile ortaya çıkar. |  |  |  |
| 22. Gıda boyaları ve koruyucular merkezi sinir sistemi problemleriyle ilişkilidir. |  |  |  |
| 23. Genetiği değiştirilmiş gıdalar çocuklarda daha az alerjik reaksiyona neden olur. |  |  |  |
| 24. Okullar ve kreşler çevresel açıdan güvenli yerlerdir. |  |  |  |
| 25. Çocuklar ev içinde dışarıdan daha yüksek konsantrasyonlarda hava kirleticilerine maruz kalırlar. |  |  |  |
| 26. Çocukların oynayabileceği park ve bahçeler çevresel kirleticilerin en az olduğu alanlardır. |  |  |  |

**ÇOCUK ÇEVRE SAĞLIĞI BECERİLERİ ANKETI**

| **Maddeler** | **1**  **Kesinlikle katılmıyorum** | **2** | **3** | **4** | **5**  **Kesinlikle katılıyorum** |
| --- | --- | --- | --- | --- | --- |
| 1. Bir çocuğun maruz kaldığı temel çevresel riskleri değerlendirebilirim. |  |  |  |  |  |
| 2. Bir çocukta solunum yolu hastalıklarına neden olabilecek çevresel riskleri belirleyemem. |  |  |  |  |  |
| 3. Bir çocukta neoplastik hastalıklara neden olabilecek çevresel riskleri belirleyebilirim. |  |  |  |  |  |
| 4. Bir çocukta nörolojik bozukluklara neden olabilecek çevresel riskleri belirleyemem. |  |  |  |  |  |
| 5. Ebeveynlere çocuklarının gıdalarındaki temel kirletici maddeler hakkında sağlık eğitimi verebilirim. |  |  |  |  |  |
| 6. Oyun alanlarındaki çevresel riskleri belirleyemem. |  |  |  |  |  |
| 7. Ebeveynlere, bir çocuğun dışarıda oynarken maruz kaldığı çevresel riskleri en aza indirecek faaliyetler hakkında sağlık eğitimi verebilirim. |  |  |  |  |  |
| 8. Bir çocuğun evindeki çevresel riskleri belirleyemem. |  |  |  |  |  |
| 9. Ebeveynlerin ev ortamındaki çevresel risklerle ilgili sağlığı geliştirme gereksinimini karşılayabilirim. |  |  |  |  |  |
| 10. Bir çocuğun okulundaki çevresel riskleri belirleyebilirim. |  |  |  |  |  |
| 11. Bir çocuğun okulunda çevresel risklerle mücadele etmek için gereken faaliyetleri belirleyemem. |  |  |  |  |  |
